# Cellulose-Based Composites as Scaffolds for Tissue Engineering: Recent Advances

**DOI:** 10.3390/molecules27248830

**Published:** 2022-12-12

**Authors:** Siavash Iravani, Rajender S. Varma

**Affiliations:** 1 Faculty of Pharmacy and Pharmaceutical Sciences, Isfahan University of Medical Sciences, Isfahan 81746-73461, Iran; 2 Regional Centre of Advanced Technologies and Materials, Czech Advanced Technology and Research Institute, Palacký University in Olomouc, Šlechtitelů 27, 783 71 Olomouc, Czech Republic

**Keywords:** scaffolds, cellulose-based scaffolds, cellulose, tissue engineering, biocompatibility, degradability

## Abstract

Today, numerous studies have focused on the design of novel scaffolds for tissue engineering and regenerative medicine applications; however, several challenges still exist in terms of biocompatibility/cytocompatibility, degradability, cell attachment/proliferation, nutrient diffusion, large-scale production, and clinical translation studies. Greener and safer technologies can help to produce scaffolds with the benefits of cost-effectiveness, high biocompatibility, and biorenewability/sustainability, reducing their toxicity and possible side effects. However, some challenges persist regarding their degradability, purity, having enough porosity, and possible immunogenicity. In this context, naturally derived cellulose-based scaffolds with high biocompatibility, ease of production, availability, sustainability/renewability, and environmentally benign attributes can be applied for designing scaffolds. These cellulose-based scaffolds have shown unique mechanical properties, improved cell attachment/proliferation, multifunctionality, and enhanced biocompatibility/cytocompatibility, which make them promising candidates for tissue engineering applications. Herein, the salient developments pertaining to cellulose-based scaffolds for neural, bone, cardiovascular, and skin tissue engineering are deliberated, focusing on the challenges and opportunities.

## 1. Introduction

**Figure 1:**
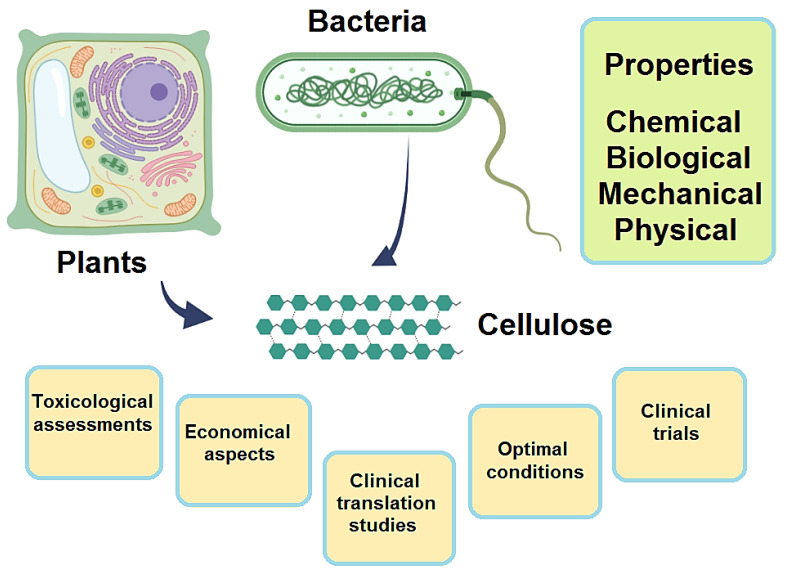
Crucial aspects and properties of cellulose-based tissue engineering scaffolds.

Cellulose with the fascinating properties of renewability, cost-effectiveness, and mechanical resilience has been widely deployed in designing composite scaffolds for tissue engineering (Figure 1) [1,2,3,4]. However, its degradation and reabsorption in tissue engineering are crucial challenges that can restrict its broader appliance in tissue engineering. To improve the catabolic and biosorption properties of cellulose in living systems, modification or functionalization ought to be specifically accomplished using various polymers, proteins, and solvents, etc. [5]. This abundant and sustainable natural material can be employed as a potential biopolymer to construct scaffolds and three-dimensional (3D) printed products instead of using non-renewable polymers [6,7,8,9]. In this context, the isolation techniques, number of inter/intramolecular hydrogen bonds, chain length, and crystallinity can affect the physicochemical properties of natural cellulose [6]. Typically, cellulose nanofibers, nanocrystals [10], and bacterial nanocellulose are the main categories of nanocelluloses with unique mechanical features and biocompatibilities for biomedical applications [11,12,13]. Different cellulose-based materials have been deployed in designing biocompatible and multifunctional scaffolds (Table 1) [14,15].

Plant biomass can be considered as the main natural source of cellulose, but cellulose can also be extracted from other natural resources such as algae, fungi, and bacteria strains [16,17]. Among them, bacterial celluloses extracellularly produced by bacteria (e.g., *Komagataeibacter xylinus* and *Gluconacetobacter xylinum*) have been widely exploited by researchers in recent years (especially for tissue engineering [18]) because of their salient advantages such as excellent biocompatibility, mouldability, biodegradability, chemical stability, liquid/gas permeability, purity, and unique mechanical properties [5,19,20,21]. These materials with different structures can be obtained using several strategies, including biological techniques, physical modifications (such as coating, doping, and blending), and chemical modifications (such as polymer grafting and molecular modification) [5]. For instance, bacterial cellulose tubes with unique mechanical features similar to porcine carotid arteries have been evaluated (in vivo) as artificial blood vessels [19].

Nanocellulose-based hydrogels are receiving immense consideration in drug delivery, tissue engineering, wound dressings, and biosensing because of their unique mechanical properties, flexibility, surface chemistry, moldability, biocompatibility, and high water-holding capacity [22,23,24]. To develop scaffolds based on nanocellulose gels and foams, structural properties (such as the porosity, mechanical features, and morphology) as well as biological interactions (especially biodegradability and biocompatibility) are very important and ought to be optimized based on specific tissues, as has been comprehensively reviewed by Ferreira et al. [25]. In addition, the microstructural analyses of cellulose-nanocrystal-based suspensions and hydrogels using imaging and rheological techniques have been deliberated in detail [26]. Using 3D bioprinting technologies, various nanocellulose-based hydrogels have been introduced for cartilage tissue engineering [27]. Hydrogels, with their robust tissue adhesion and soft mechanical features (such as unique elasticity and swelling capacity), can be applied as attractive candidates in designing tissue-engineering scaffolds. In this context, because of the suitable biodegradability and mechanical strength of hydrogels based on cellulose, they have been widely deployed in wound dressing and tissue engineering applications [28]. For instance, bacterial cellulose and silk fibroin double-network hydrogel was fabricated with a highly interconnected and open porous structure along with a high mechanical strength and biocompatibility for cartilage tissue engineering; this hydrogel was prepared by simply soaking bacterial cellulose in an aqueous silk fibroin solution with no need for any cross-linking agents [29].

**Table 1:** Some selected examples of cellulose-based scaffolds with tissue engineering applications.

Cellulose-Based Scaffolds	Applications	Advantages/Properties	Refs.
Collagen modified by 2,3 dialdehyde cellulose	Neural tissue engineering	- High surface area to pore volume ratio - The magnitude of conductivity for the collagen/cellulose composite was ~40% lower than that of pristine collagen	[30]
Bio-based cellulosic scaffold	Tissue engineering and drug delivery	- Significantly porous scaffold with robust network of ultrathin cellulosic layers - Multifunctionality with advantages of lipophilicity, hydrophobicity, and oleophilicity	[31]
Hydrosoluble phosphorous-acid-derivatized cellulose	Cell culture (in vitro) and tissue regeneration (in vivo)	- Good cytocompatibility and lack of toxicity - Enhanced bioactivity by phosphorylation	[32]
Cellulose/soy protein isolate/calcium phosphate hybrid	Tissue engineering	- Good biocompability - Biomimetic calcium phosphate mineralization	[33]
Cellulose-nanocrystal-reinforced maleic anhydride-g-poly(butylene adipate-co-terephthalate) bionanocomposites	Tissue engineering	- Improved thermal stability - Improvements in the viscoelastic features - Improvements in cell adhesion - Good biocompatibility	[34]
Cellulose nanocrystals and reduced graphene oxide into poly-lactic acid matrix nanocomposites	Antibacterial effects against *Staphylococcus aureus* and *Escherichia coli*	- Increased tensile strength - Efficient antibacterial effects - Negligible cytotoxicity	[35]
Nanocellulose and nanochitin hydrogels	Bone tissue engineering	- Biomimetic scaffolds - Good biocompatibility - Low immunogenicity	[36]
Regenerated modifiedcellulose films (micro-fibrillated cellulose)	Tissue engineering	- Improved cell attachment - Tunable attachment and scaffold mechanics	[37]
Cellulose-chitosan hydrogels	Tissue engineering	- Improved cell attachment - Increased charge density and/or shear modulus	[38]
Electrospun fiber meshes (oxidation followed by sulfonation)	Bone-tissue engineering	- High retention capacity for human recombinant bone morphogenetic protein-2 - The retained proteins could remain biologically active for at least seven days - Robust structural and mechanical integrity	[39]
Three-dimensional cellulose scaffolds (decellularization followed by glutaraldehyde cross-linking)	The culture of mammalian cells (in vitro)	- Tunable surface biochemistry and mechanical features - The cells retain high viability (after 12 weeks of culture) - Easy to produce, inexpensive, and renewable	[40]
Polydopamine on electrospun poly(lactic acid)/cellulose nanofibrils	Tissue engineering, biomimetic composite scaffolds (acceleration in cell biocompatibility)	- Improved hydrophilicity, mechanical characteristics, and biocompatibility - Improved adhesion, proliferation, and growth of human mesenchymal stem cells cultured on the scaffold	[41]
Thermoplastic polyurethane nanofiber/cellulose nanofibrils	Tissue engineering	- Improved hydrophilicity and mechanical features - Enhanced adhesion and proliferation of human umbilical vein endothelial cells cultured on the scaffold	[42]
Hydroxypropyl cellulose methacrylate	Long-term cell culture and implantable tissue scaffolds	- Good biocompatibility and biodegradability - Cytocompatibility and mechanical rigidity	[43]
Ethyl hydroxy ethyl cellulose/poly(vinyl alcohol) nanofibers	Tissue engineering and drug delivery	- Controlled release of antibacterial drugs - Good biocompatibility and nontoxicity	[44]
Macroporous hydroxypropyl cellulose methacrylate scaffold	Adipose tissue engineering	- The pore size was 30–300 μm, and the interconnected porosity was ∼90% - Good biocompatibility toward human adipose-derived stem cells - Thermal responsive phase behavior	[45]
Gelatin-carboxymethylcellulose hydrogels	Engineering vascularized and cell-dense 3D tissues/organs	- Suitable candidates for rapid preparation of perfusable vascular networks - Good cytocompatibility - Suitable microenvironment for angiogenesis	[46]
Pectin/carboxymethyl cellulose/microfibrillated cellulose composite scaffolds	Tissue engineering	- Improved thermal stability and low degradation rate - Good cytocompatibility on NIH3T3 cell lines - Controlled swelling and degradation behavior	[47]
Electrospun nanofiber constructed from cellulose acetate with polymer graft and polydopamine coating	Tissue engineering scaffolds and antibacterial effects	- Free-standing nanofiber mats with high performances - Antibacterial effects (reduction in microbial attachment) - Good stability	[48]
Sugar-cane-bagasse-derived cellulose-based electrospun nanofiber mats	Tissue engineering	- Good biocompatibility - Unique physicochemical and biological properties with enhanced performance for tissue engineering purposes	[49]
Cellulose-binding domain of the Cellulomonas fimi CenA protein	Biosensor scaffolds for fluorescence lifetime imaging-assisted tissue engineering	- Adjusted bio-formation of 3D tissue models with recognized metabolic properties - Measurement of pH and Ca2+ gradients by fluorescence intensity and lifetime imaging detection modes	[50]
α-cellulose-epoxidized soybean oil scaffolds	Tissue engineering	- Good biocompatibility (in vitro) - Good surface and internal structures for homogeneous cell attachment and growth - Multi-scale porosity for tissue engineering applications	[51]

Cellulose-based materials with environment-friendly properties have been widely employed for the low-cost manufacturing of tissue engineering scaffolds [52,53,54]. However, for success in tissue regeneration, crucial factors, namely cell adhesion, biological signaling, and cell responsive degradation, ought to be considered. One of the main challenges in tissue engineering is the introduction of suitable substrates for supporting stem cell growth and proliferation to efficiently repair the damaged tissues [55]. Notably, designing scaffolds utilizing plant tissues can be considered as an alternative for extracellular matrices (ECM). For instance, decellularized onion scaffolds with unique 3D structures, interconnected pores, and moderate surface roughness were designed for supporting osteogenic differentiation [55]. Accordingly, studies revealed that alkaline phosphatase activity and calcium deposition in human mesenchymal stem cells differentiated on these scaffolds were considerably higher than cells distinguished on tissue culture polystyrene (the control group); the expression level of common bone-related genes in human mesenchymal stem cells was also highly improved compared to the cells cultured in the control group. These decellularized onion scaffolds can be considered as promising supportive materials for stem cell proliferation/differentiation in tissue engineering due to their cost-effectiveness and environmentally benign attributes [55]. Another crucial aspect is the rapid degradation of natural polymers, which may restrict their practical tissue engineering applications; ideal scaffolds should have enough capability for repairing body tissues mimicking the features of ECM of tissues for regeneration with appropriate degradation during or after the healing process [56,57,58,59,60]. The properties of scaffolds can be improved by designing hybrid composite scaffolds using different polymers or via functionalization with suitable biocompatible/bioactive agents [52,61]. Notably, the biological characteristics of scaffolds can significantly affect the interaction of scaffolds with organs/tissues; thus, further explorations ought to be focused on the incorporation of bioactive scaffolds to promote appropriate cellular interactions and migration/differentiation [59]. This review not only summarizes recent studies on developing cellulose-based scaffolds for tissue engineering but also deliberates the crucial challenges and prospects resulting from the expansion in application of these composite scaffolds in the realm of neural, bone, cardiovascular, and skin tissue engineering.

## 2. Cellulose-Based Scaffolds for Tissue Engineering

Several cellulose-based composite scaffolds have been introduced with unique architectures, surface chemistry, and excellent cell attachment/proliferation [62,63]. However, additional explorations still need to center around comprehensive evaluations of their degradability, long-term biosafety, and possible immunogenicity [64].

### 2.1. Neural Tissue Engineering

Typically, biomaterials applied for neural tissue engineering for restoring lost functions in nervous systems should have biocompatibility, flexibility, a suitable degradation rate, longitudinal channels for enhancing the regeneration of axons, and bioactivity. To overcome biological restraints on neural regeneration and restoration, these biomaterials should have enough capabilities for cellular growth and behavior [65]. In a study pertaining to design neural tissue engineering scaffolds, conductive polypyrrole on electrospun cellulose nanofibers were synthesized [66]. After culture studies (in vitro) on SH-SY5Y human neuroblastoma cells, it was revealed that improved cell adhesion on the scaffold could be attained [66]. In addition, 6-carboxycellulose was prepared for tissue engineering purposes [67]. Accordingly, after the functionalization of cellulose with arginine or chitosan, the phenotypic maturation of vascular smooth muscle cells could be improved; chitosan could improve the adhesion and growth of these cells [67].

Cellulose/conductive polymer nanofibrous mats comprising electrospun cellulose/poly *N*-vinylpyrrole and electrospun cellulose/poly(3-hexylthiophene) have been fabricated using an in-situ polymerization technique [68]. These composite mats exhibited enhanced thickness and conductivity along with the improved porosity. After cytocompatibility studies (in vivo) on undifferentiated PC12 cells, it was revealed that these scaffolds had suitable cell activity, proliferation, and adhesion. The electrospun cellulose/poly(3-hexylthiophene) mats promoted the proliferation of the PC12 cells more than the corresponding electrospun cellulose and cellulose/poly *N*-vinylpyrrole mats [68]. Nanocrystalline cellulose hydrogels were designed using bacterial cellulose (from *Acetobacter xylinum*) for 3D neuronal bilayer generation, providing novel hydrogels for neural engineering applications and neurobiology explorations [69]. Graphene oxide nanoflakes were introduced into bacterial cellulose culture media to induce the structural modifications within the crystalline cellulose nanofibrils and to modulate their 3D collective associations, causing a considerable reduction in Young’s modulus and the clear definition of water–hydrogel interfaces. Accordingly, enhanced neurite outgrowth with a decreased backward travel length along with the suitable generation of synaptic connectivity with distinct axonal bifurcation abundancy could be obtained [69].

### 2.2. Bone and Cartilage Tissue Engineering

Various cellulose-based scaffolds have been designed with excellent potential for bone tissue engineering [70]; however, before clinical translation, challenges associated with the flaws in the currently applied preclinical models still exist [70]. In addition, other important arguments in designing polymeric scaffolds for bone tissue engineering are the lack of osteoconductivity as well as the risk of inflammatory reactions caused by the degraded by-products [71,72]. In one study, 3D porous scaffolds were prepared from cellulose using non-hydrolytic sol–gel and lyophilization approaches. Afterward, cuttlebone microparticles were immobilized to stimulate the osteoconductive features of the polymeric scaffolds, and the surface coating could be obtained through in-vitro mineralization using 10-fold concentrated simulated body fluid. Scaffolds with improved cell attachment and suitable proliferative/osteoconductive effects on osteoblast-like MG-63 cells are considered as promising candidates for bone tissue engineering [71].

**Figure 2:**
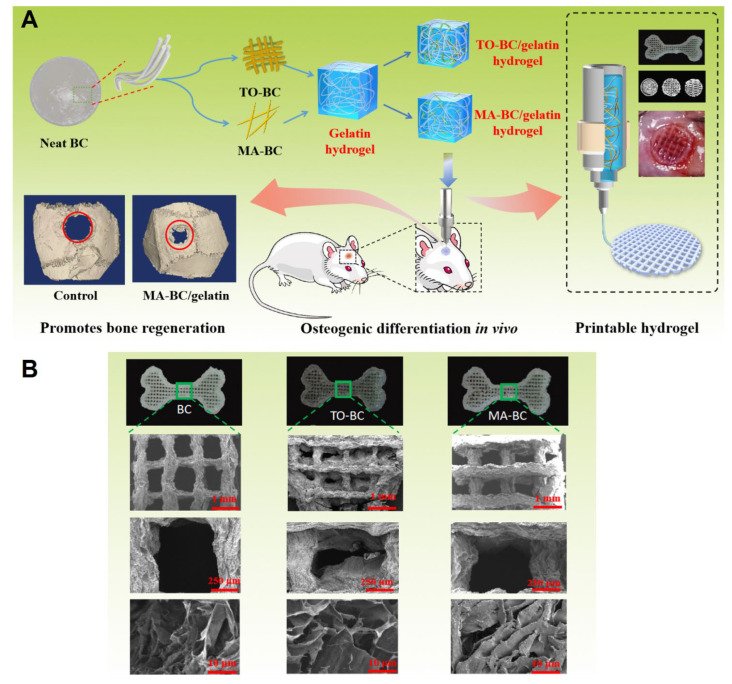
(**A**) The preparative process of printable bacterial cellulose–gelatin gels for osteogenic regeneration (in vivo). (**B**) Scanning electron microscopy (SEM) images of printed scaffolds (BC, TO-BC, and MA-BC). Adapted from reference [74] with permission. Copyright 2021 Elsevier.

He et al. [73] designed silk fibroin/cellulose nano whiskers-chitosan composite scaffolds through a layer-by-layer assembly technique, which provided unique mechanical properties and good biocompatibility. These scaffolds could successfully support the proliferation of cells and stimulate the levels of biomineralization-relevant alkaline phosphatase activity and osteocalcin expression, thus exhibiting suitable applicability for bone implantation and generation [73]. In another study, for the purpose of fabricating bone tissue engineering scaffolds, neat bacterial cellulose was treated with TEMPO (2,2,6,6-tetramethylpiperidine-1-oxyl radical)-mediated oxidation (TO-BC) and maleic acid (MA-BC) to acquire homogeneous bacterial cellulose dispersions (Figure 2) [74]. Accordingly, the hybridization of MA-BC with gelatin was performed, providing a gel with superior rheological features and a superior compression modulus for 3D printing. Both the prepared dispersions exhibited suitable osteoblast viability, but MA-BC revealed an improved capability for expressing osteogenic marker genes and forming mineralized nodules (in vitro). In addition, the MA-BC-based gelatin scaffolds displayed improved capabilities for stimulating rat calvaria regeneration compared to the TO-BC ones, thus presenting a better bone mineral density of the newly formed bone and a better trabecular thickness (in vivo) [74].

Hydrogel scaffolds with good biocompatibility (in vitro) and bubble-like porous structures were fabricated using hydroxyethyl chitosan and cellulose. These hybrid scaffolds could efficiently support the attachment and proliferation of osteoblastic MC3T3-E1 cells, which introduced them as attractive candidates for bone tissue engineering [75]. In addition, calcium-filled bacterial-cellulose-based hydrogel scaffolds have been fabricated for bone tissue engineering, providing excellent cell growth and proliferation [76]. Among the introduced hydrogel scaffolds in this study, bacterial cellulose-polyvinylpyrrolidone-*β*-tricalcium phosphate/hydroxyapatite with notable cytocompatibility/biocompatibility displayed excellent potential to facilitate musculoskeletal (bio)engineering [76].

**Figure 3:**
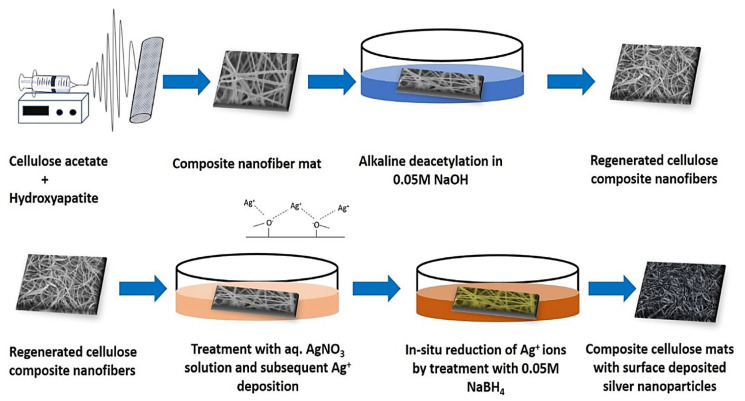
The preparative process of regenerated cellulose nanofibers from cellulose acetate and incorporation of hydroxyapatite and silver (Ag) nanoparticles in designing tissue engineering scaffolds. Adapted from reference [77] with permission. Copyright 2020 Elsevier.

Acetate-free nanofibers were synthesized by the alkaline de-acetylation of as-spun nanofibers (Figure 3) [77]. These cellulose nanofibers loaded with hydroxyapatite were immobilized with the deployment of silver nanoparticles to produce nanofiber scaffolds for wound healing and bone tissue engineering. These nanofibers with good cytocompatibility exhibited suitable antimicrobial effects against *E. coli* and *S. aureus*, opening unlimited opportunities for soft- and hard-tissue engineering with cell proliferation and antibacterial benefits [77]. Since pristine cellulose cannot have antibacterial effects, it should be combined with other materials to augment its antimicrobial properties.

For cartilage tissue engineering, scaffolds should have high porosity and pore-to-pore inter-connectivity as well as enough space for in vitro cell adhesion, in-growth, and the rearrangement of cells. Interconnected porous organization can facilitate the migration of cells, the spread of physiological nutrients/gasses to cells, and the release of metabolic waste and by-products from the cells [22]. In one study, after the addition of azide and alkyne moieties to citric-acid-modified hydroxyethyl cellulose structures, crosslinked cellulose-based scaffolds could be obtained through the 2022 Nobel-award-winning bio-orthogonal click chemistry technique, including strain-promoted azide-alkyne cycloaddition [78]. These scaffolds with porous interconnected microarchitectures exhibited unique properties such as improved stability, extensive water uptake, and a swelling degree of ~650%, which made them suitable for cartilage tissue engineering. In addition, the mechanical properties of these scaffolds with a tensile strength of ~0.43 MPa and Young’s modulus of ~10 Mpa, as well as their biocompatibility, chondrogenic ability, and bio-orthogonal properties, were comparable with those of normal cartilage tissue [78].

Starch/cellulose nanofiber composites were designed with enhanced biodegradability, porosity, and mechanical strength for cartilage tissue engineering [79]. An enhancement in pore interconnectivity could be achieved after increasing the ratio of sodium chloride in the salt leaching. The scaffolds showed adequate mechanical properties for cartilage tissue engineering applications. The water uptake ratio of the composites could be vastly increased through the addition of 10% cellulose nanofibers. The scaffolds made of starch/cellulose nanofibers were partially destroyed owing to the low degradation rate (in vitro) even after >20 weeks. The incorporation of nanofibers in the starch structure improved the cell proliferation/attachment after studies on the cultivation of isolated rabbit chondrocytes on these scaffolds [79].

**Figure 4:**
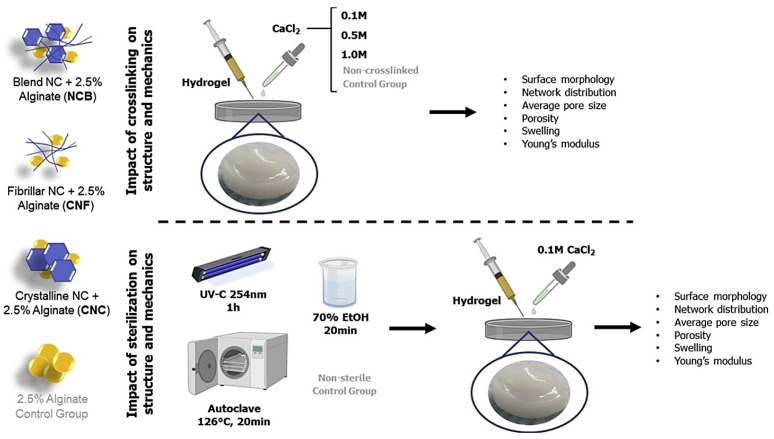
The impact of crosslinking and sterilization on mechanical and structural properties of nanocellulose-based hydrogels for cartilage tissue engineering. NCB: nanocellulose blend; CNC: nanocellulose crystals; CNF: nanocellulose fibrils. Adapted from reference [27] with permission. Copyright 2019 Elsevier.

It was revealed that the concentration of calcium chloride crosslinkers and sterilization techniques could affect the structural and mechanical features of scaffolds applied in cartilage tissue engineering, as exemplified in one study, wherein nanocellulose-based hydrogels comprising plant-derived cellulose nanofibrils and cellulose nanocrystals were evaluated (Figure 4) [27]. Therefore, crosslinking could highly modify the overall network distribution, surface morphology, pore size and porosity of the hydrogels; by increasing the concentration of CaCl_2_, an organized network in the hydrogels could be promoted. The sterilization technique could also affect the pore size and swelling of hydrogels; all the introduced sterilization techniques could structurally alter the alginate and nanocellulose-based hydrogels. In addition, ethanol sterilization could improve the mechanical features of the alginate, nanocellulose crystal, and nanocellulose fibrils. Notably, autoclaving was suggested as the optimal technique for ensuring the removal of possible contaminants [27].

### 2.3. Cardiovascular Tissue Engineering

**Figure 5:**
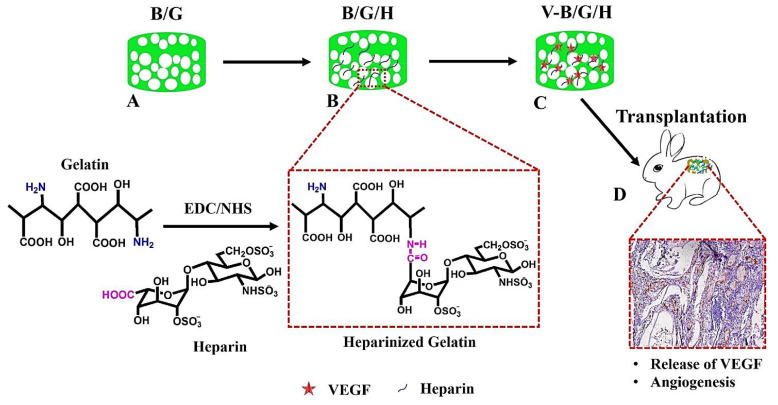
(**A**–**C**) The preparative strategy of VEGF-loaded 3D porous bacterial cellulose/gelatin scaffolds modified with heparin for vascularization and tissue regeneration purposes. After the condensation of the -COOH in the heparin and the -NH_2_ in the gelatin, the scaffolds could be prepared for (**D**) subcutaneous transplantation to provide enhanced angiogenesis. NHS: *N*-hydroxysulfosuccinimide; EDC: 1-Ethyl-3-(3-dimethylaminopropyl) carbodiimide hydrochloride; B/G: bacterial cellulose/gelatin; H: heparin. Adapted from reference [80] with permission. Copyright 2017 Elsevier.

Vascularization is an important criterion in tissue engineering/regeneration, as it affects the long-term survival of scaffolds [80]. In this context, the delivery of angiogenic factors is vital during the regeneration process to develop appropriate vascular networks. In one study, vascular-endothelial-growth-factor (VEGF)-loaded 3D porous bacterial cellulose/gelatin scaffolds were designed and modified with heparin, providing a prolonged release of VEGF for about 2 weeks (Figure 5) [80]. After cellular evaluations (in vitro), it was revealed that both migration and proliferation could be stimulated in the presence of VEGF. The angiogenesis could be highly enhanced after subcutaneous implantation, showing the excellent potential of the heparinized scaffolds loaded with VEGF for tissue regeneration; however, the next step should focus on comprehensive in vivo and clinical assessments [80].

Biocompatible cellulose-based scaffolds were developed using microcrystal cellulose and a cellulose whisker through deacetylation and electrospinning techniques for vascular-tissue-engineering applications [81]. The addition of microcrystal cellulose and cellulose whiskers to the cellulose acetate scaffold could improve the cell attachment/proliferation because of the high porosity and surface roughness of the fibers along with the non-cytotoxicity of the cellulose whiskers [81]. In addition, cardiac patches were designed utilizing cellulose acetate and regenerated cellulose, indicating suitable electromechanical functions [82]. The cellulose acetate was partially deacetylated and hydrolyzed to form regenerated cellulose and enhance the porosity of the produced scaffolds. These scaffolds exhibited improved cell growth and connectivity owing to the cell compatibility of cellulose acetate and the resemblance of a polysaccharide scaffold microenvironment to the natural cell ECM, which rendered them attractive candidates for cardiac tissue engineering [82].

For preventing myocardial post-infarction pathology, bacterial cellulose membrane patches containing co-cultured cells were designed [83]. Accordingly, the co-cultured cells retained a viability of >90% over 14 days in a culture; these patches were deployed to the myocardial surface of the infarcted area after staying for 14 days in the culture. The bacterial cellulose membrane without cellular treatment displayed a higher preservation of the cardiac dimensions. Bacterial cellulose supported the cells to generate cardio-protective soluble factors, offering patches with efficient therapeutics for patients with ischemic heart disease [83]. In addition, bacterial cellulose (BASYC^®^) has been introduced for the implantation and long-term maintenance of carotid arteries in animals (rats and pigs) [84].

Andrade et al. [85] developed the fabrication of chimeric proteins consisting of a cellulose-binding module and an adhesion peptide for enhancing the adhesion of human microvascular endothelial cells to bacterial cellulose; the recombinant proteins containing adhesion sequences with significant affinity and specificity were able to enhance the attachment of human microvascular endothelial cells to bacterial cellulose surfaces [85]. In addition, tissue-engineered vascular grafts with a length of 20 cm and an inner diameter of 3 mm were designed from bacterial cellulose for endothelialization and specific surgical features (in vivo) [86]. After the implantation of the vascular graft as an aortocoronary bypass in a left anterior descending occluded pig model, an excellent potential of small-diameter bacterial cellulose grafts for coronary and peripheral bypass grafting could be achieved [86]. Liu et al. [87] constructed novel composites from hierarchical-structured bacterial cellulose and potato starch, which showed good biocompatibility for vascular tissue engineering. The bacterial cellulose/potato starch tubes exhibited promising capabilities as artificial small-diameter vascular grafts These grafts with dense inner surfaces and circumferential macroporous outer layers could rapidly promote the regeneration of blood vessels with complete endothelium monolayers, organized smooth muscle cells, rich new capillaries, and a deposited extracellular matrix in vivo [87].

### 2.4. Skin Tissue Engineering

Limited studies have been undertaken on the skin-tissue-engineering applications of cellulose-based composites. In one study, ulvan-cellulose scaffolds were synthesized via skin tissue engineering that were endowed with good biocompatibility (in vivo) and that exhibited enhanced cell growth and angiogenesis [88]. Additionally, sugar-cane-bagasse-cellulose-based scaffolds were designed for tissue healing and regeneration purposes as well as for their supportive potential for cell growth. These scaffolds with good biocompatibility have shown great potential in mimicking the in vivo setting of skin repair and regeneration, providing scaffolds with wound healing abilities [14]. To attain success in chronic and burned wound dressings, scaffolds should have enough mechanical resistance, good biocompatibility, stimulatory capability for healing, antibacterial effects, and the ability to prevent fluid loss [89]. Furthermore, keratin/bacterial-cellulose-based scaffolds were designed for burn wound dressing with nontoxicity (in vitro). Thus, the healing process by necrotic tissue detersion and the reconstruction of damaged structures using heat agents could be obtained faster and better than the control, thus revealing the promising capacities of these cellulose-based scaffolds containing stem cells for wound dressing [89].

## 3. Challenges and Future Perspectives

Cellulose-based scaffolds have been extensively designed for tissue regeneration and engineering applications [28,90]. However, crucial aspects regarding the immunogenicity, degradation time, and possible side effects still need to be considered for in vivo and clinical assessments [91]. Remarkably, the physicochemical (e.g., chemical composition, size/morphology, and porosity), mechanical (e.g., Young’s modulus, storage/loss modulus, and compressive stress), biological (e.g., immunogenicity, histology, vascular ingrowth, biocompatibility, and in vitro/in vivo analyses), diffusion (e.g., diffusion of growth factors, nutrients, and gases), and degradation (e.g., swelling behaviors, enzymatic degradation, and degradation rate) properties are vital parameters in the assessment of cellulose-based scaffolds [92]. The relationship between the mechanical features, geometries, and biological properties of scaffolds in tissue engineering ought to be further explored [93]. On the other hand, the advantages comprising environmentally benign synthesis techniques with cost-effectiveness and low energy consumption can help to generate scaffolds with commercial potential. The next steps should be planned for the translation of the lab-scale construction of scaffolds into large-scale production, focusing on the optimization of conditions along with the deployment of suitable functionalization strategies [94]. Another important challenge is designing a scaffold with optimal stimuli capabilities for supporting cell differentiation/proliferation, forming appropriate ECM components, and releasing enzymes to alter the ensuing ECM [95].

A wide variety of tissue regeneration techniques have been introduced for clinically treating damaged organs/tissues using scaffolds, but still some challenging issues need to be addressed such as insufficiency in mechanical strength, lack of vascularity, biocompatibility, degradation/resorption kinetics, nutrient diffusion, and cell proliferation/tissue growth rate [96,97,98]. In addition, tissue engineering scaffolds with the delivery potential for growth factors, cytokines, and adhesion peptides have received special attention from researchers [53]. In addition, the incorporation of anti-inflammatory and antimicrobial agents in these scaffolds can be applied as suitable strategies for reducing the infection possibility after surgical procedures. These scaffolds can be employed for the delivery of therapeutic genes using innovative gene therapy tactics with the utilization of DNA encoding for therapeutic genes, which can help to assist in the controlled/sustained release of therapeutic factors, thus enhancing the healing process [99,100].

Overall, several physicochemical features can affect the cell adhesion and proliferation in tissue-engineering scaffolds [101]. Indeed, cell attachment is affected by various factors such as material surface characteristics, environmental parameters, and cell behavior. Surface hydrophobicity, protein adsorption, surface charge, and surface softness/stiffness/roughness should be considered as the crucial factors influencing cell adhesion and behavior [102,103]. Notably, one of the vital challenges in the application of tissue engineering scaffolds is the rapid cell attachment/proliferation on the outer edge of the scaffolds, thus restricting the penetration of cells to the center of the scaffolds and forming a necrotic core. In in vitro tissue engineering studies, this challenge can be addressed by modifying the culture conditions applied for growing the tissue. In addition, designing optimized scaffolds with improved capabilities can help in transferring nutrients and cells to the center (in vitro and in vivo) [102]. On the other hand, mechanical properties and geometry are decisive scaffold properties that can affect the applicability of scaffolds and cell attachment [104]. Several geometric factors such as pore size, porosity, and connectivity/tortuosity affecting nutrient transport and cell ingrowth should be tuned for specific cells or tissues [105]. However, several inconsistent factors exist that may restrict the assessment of the scaffold design, including porosity with strength, fatigue life, and pore size with surface area; thus, a large number of in vivo analyses are essential in designing an optimized scaffold [106].

Compared to animal-derived or synthetic materials, cellulose-based materials, with their unique attributes of inexpensiveness, renewability, and environmentally benign nature, can be considered as promising alternatives for designing future tissue engineering scaffolds; synthetic biomaterials produced by chemical processes may increase the utilization of perilous agents as well as the formation of unwanted/hazardous by-products [53]. Despite the salient advantages and properties of cellulose-based scaffolds, their large-scale production, commercial applications, biodegradation, and clinical translation still face challenges [107]. In this context, specific and comprehensive in vitro (such as cell culture, seeding, attachment/viability, and distributions) and in vivo/clinical trials assessments are vital in designing optimized scaffolds [106]. Notably, the bioadaptability of biomaterials related to the properties and biological features of the materials is an important aspect. The microenvironment formed by biomaterials should be adaptable to the native microenvironment (in situ), and also their mechanical features need to be adaptable to the native tissue; the degradation features of biomaterials are other important criteria that should be adaptable with the new tissue creation [108,109].

## 4. Conclusions and Future Outlooks

Cellulose-based composites have garnered immense attention from researchers in recent years due to their high biocompatibility, nontoxicity, sustainability, and biodegradability advantages. Cellulose as a renewable and abundant material has been broadly utilized in designing composite scaffolds with tissue regeneration and engineering applications. In this context, safer and greener technologies with the utilization of safer solvents/auxiliaries can enhance the biosafety and biocompatibility properties of scaffolds, thus reducing their possible side effects in tissue engineering. Finding optimal synthesis and functionalization conditions can help assist in the design of cellulose-based scaffolds with improved functionality, stability, degradability, and biocompatibility. Although plant and bacterial celluloses have several similarities in terms of chemical structure, bacterial celluloses show some salient advantages such as higher flexibility and purity, which make them promising candidates for tissue engineering. The mechanical features (especially mechanical strength) of cellulose-based scaffolds are very important and should be optimized for their future clinical and biomedical applications. Despite various toxicological and biosafety/biocompatibility analyses of cellulose-based scaffolds, the next steps should embark on their long-term biosafety, systemic toxicity, and immunogenicity/hemocompatibility along with clinical translation studies and clinical trials. Several sterilization techniques such as ultraviolet or gamma radiation, argon plasma, or autoclaving have been introduced, but still more evaluations are required to uncover an efficient method that is without effects on the structural and functional properties of scaffolds.

## Data Availability

Not applicable.
